# PIWI-interacting RNA-YBX1 inhibits proliferation and metastasis by the MAPK signaling pathway via YBX1 in triple-negative breast cancer

**DOI:** 10.1038/s41420-023-01771-w

**Published:** 2024-01-05

**Authors:** Linyu Wu, Shanshan Huang, Wenwen Tian, Peng Liu, Yi Xie, Yu Qiu, Xing Li, Yuhui Tang, Shaoquan Zheng, Yuying Sun, Hailin Tang, Wei Du, Weige Tan, Xinhua Xie

**Affiliations:** 1 State Key Laboratory of Oncology in South China, Guangdong Provincial Clinical Research Center for Cancer, Sun Yat-Sen University Cancer Center, Guangzhou, 510060 China; 2 Affiliated Cancer Hospital & Institute of Guangzhou Medical University, No.78 Hengzhigang Road, Guangzhou, 510095 China; 3 Department of Breast Surgery, Breast Disease Center, The First Affiliated Hospital, Sun Yat-sen University, Guangzhou, 510080 China; 4 Department of Pathology, Changde Hospital, Xiangya School of Medicine, Central South University, Changde, 415003 China; 5 Department of Breast Surgery, The First Affiliated Hospital of Guangzhou Medical University, Guangzhou, 510120 China

**Keywords:** Cancer genetics, Cell migration

## Abstract

Breast cancer is the second leading cause of death in women worldwide, with triple-negative breast cancer (TNBC) having the worst prognosis. Although there are numerous studies on TNBC, there is no effective treatment for it, and it is still a major problem today. Studies on PIWI-interacting RNAs (piRNAs) are increasing and investigating the mechanism of piRNAs in the proliferation and metastasis of TNBC may lead to new potential treatment targets. Here, we identified a novel piRNA, piR-YBX1, which was downregulated in TNBC compared to matched normal breast tissue. Overexpression of piR-YBX1 significantly inhibited the proliferation, migration, invasion ability of TNBC cells both in vivo and in vitro. Mechanistically, piR-YBX1 could bind directly to mRNA of Y-box binding protein 1 (*YBX1*) and overexpression of piR-YBX1 downregulated YBX1 in both mRNA and protein levels, while the function of piR-YBX1 could be partly rescued by overexpression of YBX1. In addition, YBX1 could bind to RAF1 which is the key molecule in the MAPK signaling pathway, and overexpression of piR-YBX1 inhibited the p-MEK and p-ERK1/2, which can be reverted by YBX1. In conclusion, our findings discovered that the piR-YBX1/YBX1/MAPK axis suppresses the proliferation and metastasis of TNBC and therefore piR-YBX1 has the potential to be an effective therapeutic agent for breast cancer.

## Introduction

Breast cancer (BC) is a serious threat to the life and health of women worldwide. Currently, BC is the most diagnosed tumor in women and results in the second cause of death in women [1]. According to the latest data, among American women, the incidence rate of BC ranks first with 31%, and the mortality rate ranks second with 15% [2]. Based on the molecular expression patterns of BC cells, BC can be divided into three subtypes, with triple-negative breast cancer (TNBC) lacking expression of estrogen receptor (ER), progesterone receptor (PR), and human epidermal growth factor receptor 2 (HER2) expression [3, 4]. Because of lack of targets, the treatment of TNBC relies on surgery, chemotherapy, radiotherapy, and immunotherapy [5–7]. TNBC accounts for only 15% of all cases of the breast cancer [8], but has the worst prognosis, with 40% of patients dying within five years after diagnosis [9, 10]. Metastasis of the primary tumor is the leading cause of death in patients with TNBC, with the lung, bone, and liver being the top 3 organs affected. Although there are many different views to explain the mechanism of TNBC metastasis [11] and progress in looking for new treatment is going on [12, 13], the result is not satisfactory. Therefore, it is vital and urgent to explore the underlying mechanism of metastasis in TNBC and identify novel therapeutic targets.

For decades, scientists have known that only approximately 1.2% of genes can encode proteins while 80% of genes are transcribed into noncoding RNAs (ncRNAs) [14], which play significant roles in many aspects [15–17]. Besides, the function of ncRNAs not only in tumorigenesis and tumor development has been investigated in recent years [18–21], but also in the prediction of tumor occurrence and development [22]. According to their length, ncRNAs are classified into long ncRNAs and short ncRNAs, and PIWI-interacting RNAs (piRNAs) are short RNAs with 21–33 nucleotides [23]. The genomic loci generating piRNAs is called piRNA clusters, but the biogenesis of piRNAs is not clear now [24]. Some studies have reported that the number of piRNAs in the human genome is significantly higher than that of microRNAs [25–27], hence, it is worthwhile to explore the functions of piRNAs in human cancer. Although several studies have reported that piRNAs effected the progression of cancers, such as breast cancer, colorectal cancer, cervical cancer, and diffuse large B-cell lymphoma [28–31], studies examining the mechanism of piRNAs in TNBC metastasis are lacking. Therefore, it is important to further explore the function of piRNAs and their mechanism in TNBC.

In this study, we identified a novel piRNA (piR-YBX1) that is downregulated in TNBC tissue compared to matched normal breast tissue. We have investigated the inhibitory effect of piR-YBX1 on the proliferation and metastasis of TNBC cells in vitro and in vivo. Further investigation revealed that Y-box binding protein 1(*YBX1*) is the target gene of piR-YBX1, which suppress the MAPK signaling pathway. Moreover, we discovered that agopiR-YBX1 inhibits tumor development in mice, indicating that piR-YBX1 has therapeutic application potential.

## Results

### Low expression of piR-YBX1 is identified in TNBC

**Fig. 1:**
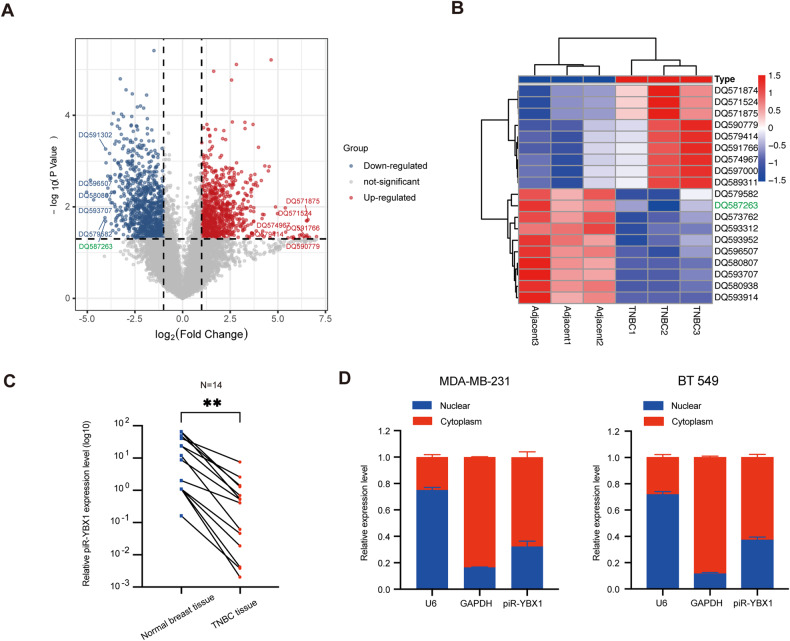
Low expression of piR-YBX1 is identified in triple-negative breast cancer (TNBC). **A** The volcano plot shows the differently expressed piRNAs. **B** The cluster heatmap shows that DQ587263 (piR-YBX1) was downregulated in TNBC tissue compared with normal breast tissue. Red and blue indicate upregulated and downregulated piRNAs, respectively. **C** The expression level of piR-YBX1 in TNBC tissue and adjacent normal breast tissue by RT–qPCR. **D** Distribution of piR-YBX1 in MDA-MB-231 and BT549 cells via nucleocytoplasmic separation assay. The data are showed as the mean ± SD, *P < 0.05 **P < 0.01, ***P < 0.001, ****P < 0.0001. Data were analyzed by (**C**) Students t test.

Three pairs of TNBC tissue and matched adjacent normal breast tissue were collected to obtain profiles of differentially expressed piRNAs using piRNA microarray, which was performed by Aksomics Inc. (Kangchen Bio-tech, China). We set the filtering criteria as a fold change of ≥2.0 and a *P*-value of <0.05, and the differently expressed piRNAs are shown in the volcano plot (Fig. 1A). Representative upregulated and downregulated piRNAs are shown in the cluster heatmap (Fig. 1B). DQ587263 (piR-54375) was one of the most strongly downregulated piRNAs in TNBC tissue and it is related to YBX1 which is a famous oncogene according to the piRBase website (http://bigdata.ibp.ac.cn/piRBase), so we named DQ587263 as piR-YBX1. Then RT–qPCR was performed to verify the expression level of piR-YBX1 in clinical samples, and the results revealed that the expression of piR-YBX1 in TNBC was lower than matched normal breast tissue (Fig. 1C). Moreover, to further determine the distribution of piR-YBX1 in breast cells, RT–qPCR after nucleocytoplasmic separation assay was undertaken which suggested that piR-YBX1 was mostly distributed in the cytoplasm (Fig. 1D).

### piR-YBX1 acts as a tumor suppressor in TNBC in vitro

**Fig. 2:**
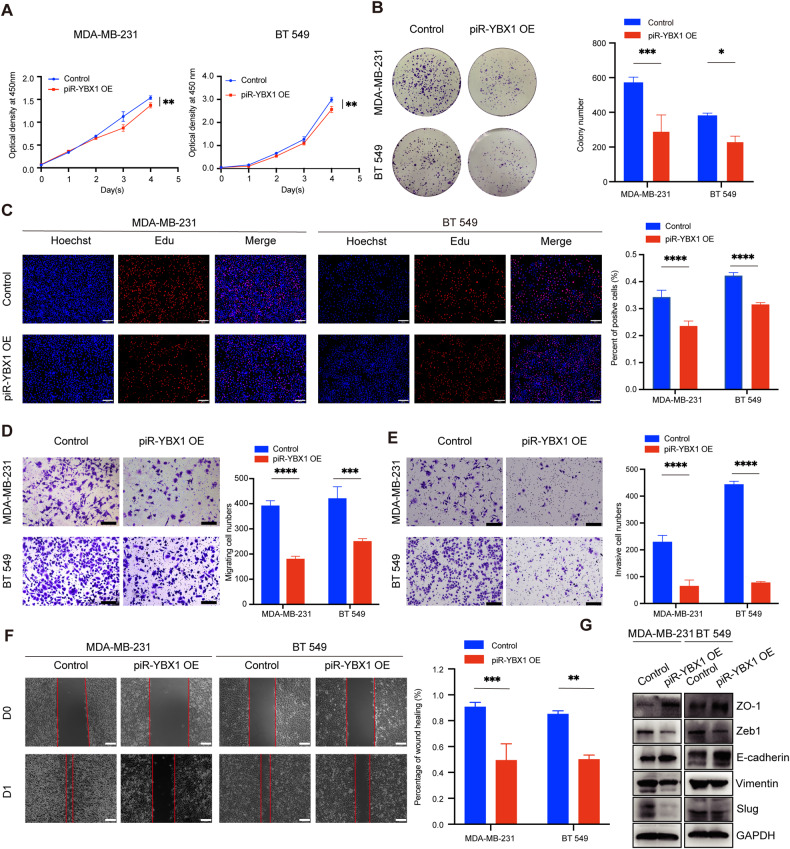
piR-YBX1 acts as a tumor suppressor in TNBC in vitro. **A** The effect of piR-YBX1 on cell proliferation was measured by a CCK8 assay. **B** A colony formation assay was utilized to measure cell proliferation. Representative pictures (left) and quantitative statistics (right) of colony numbers. **C** An EdU incorporation assay was used to assess the proliferation ability of TNBC cells. Representative pictures (left) and quantitative statistics (right) of proliferative cells. Scale bar: 100 μm. **D, E** Transwell and invasion assays were performed to evaluate the ability of migration and invasion abilities of TNBC cells. Representative pictures (left) and quantitative statistics (right) of penetrating cells. Scale bar: 100 μm. **F** A would healing assay was performed to analyze the migration ability of TNBC cells. Representative pictures (left) and quantitative statistics (right) of the migration rate. Scale bar: 100 μm. **G** The expression levels of EMT-related markers were analyzed by western blot. The data are showed as the mean ± SD, *P < 0.05 **P < 0.01, ***P < 0.001, ****P < 0.0001. Data were analyzed by (**A**) two-way ANOVA and (**B**–**F**) Students t test.

Since piR-YBX1 expression is decreased in TNBC, we explored whether it has negative effect on the malignant phenotype. Overexpression of piR-YBX1 substantially inhibited the proliferation of MDA-MB-231 and BT549 cells in the CCK8 assay (Fig. 2A). Similarly, we found that piR-YBX1 overexpression suppressed colony formation compared with that in the control group (Fig. 2B). An EdU assay was used to monitoring cellular proliferation, and the percentage of proliferative cells decreased in the overexpression group (Fig. 2C). We further detected the function of piR-YBX1 in metastasis of MDA-MB-231 and BT549 cells. The migration and invasion assays revealed that the migration and invasion abilities of MDA-MB-231 and BT549 cells were markedly reduced when piR-YBX1 was overexpressed (Fig. 2D, E). Similar results were observed in the wound healing assay, cells overexpressing piR-YBX1 had a lower migration rate than the control cells (Fig. 2F). Furthermore, we texted whether overexpression of piR-YBX1 in SUM-159-PT cells could also repress the proliferation and migration ability by CCK8 assay and transwell assay. The results demonstrated that overexpression of piR-YBX1 is sufficient to inhibit the growth and metastasis capacity in SUM-159-PT (Additional file 4: Fig. S1A, B). Because the metastatic ability of epithelial tumor cells is usually caused by EMT, we performed western blot to examine the protein levels of EMT makers. The levels of E-cadherin and ZO-1 increased, while those of Zeb-1, Vimentin, and Slug decreased in the piR-YBX1 overexpression group (Fig. 2G).

Overall, these observations demonstrated the powerful ability of piR-YBX1 to restrain the proliferation, migration, and invasion of TNBC cells.

### Negative effect of piR-YBX1 on YBX1 expression via targeting *YBX1* mRNA

**Fig. 3:**
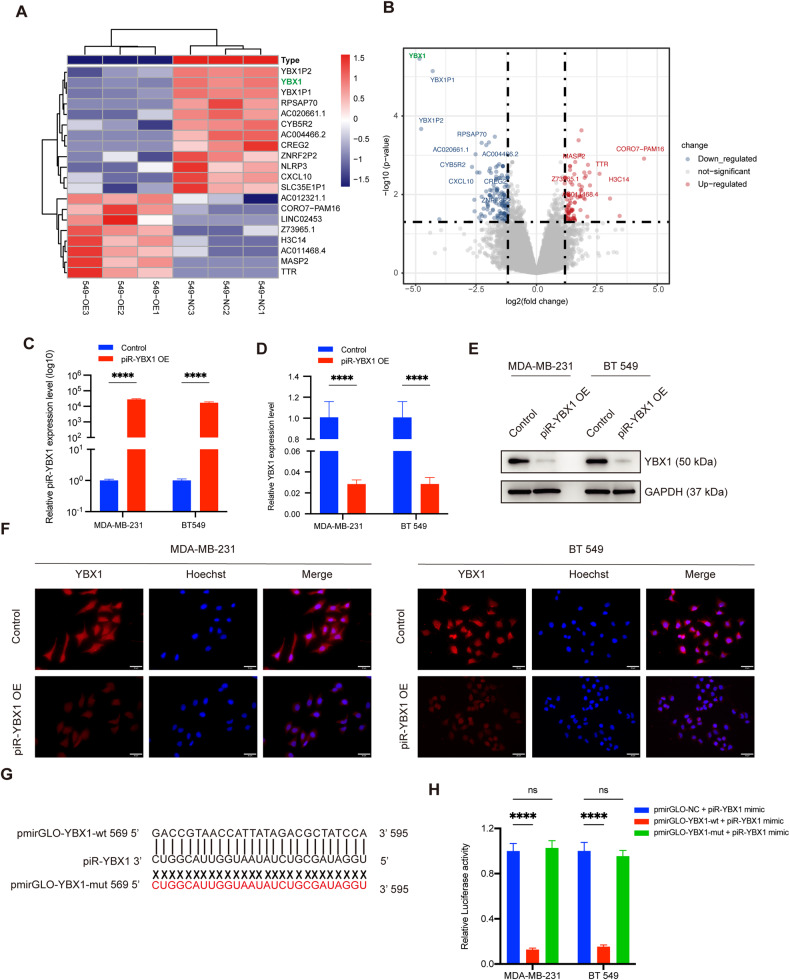
Negative effect of piR-YBX1 on Y-box binding protein 1 (YBX1) expression via targeting *YBX1* mRNA. **A** The cluster heatmap shows the top 20 changed genes in the RNA-seq. Red and blue indicate upregulated and downregulated genes, respectively. **B** The volcano plot indicates that *YBX1* is the most downregulated gene. **C** The level of piR-YBX1 in TNBC cells with stable overexpression (OE) of piR-YBX1 and control cells was measured by RT–qPCR. **D, E** Levels of *YBX1* mRNA and protein were measured by RT–qPCR and western blot, respectively, in piR-YBX1 OE or control TNBC cells. **F** An immunofluorescence assay was used to show the change in the YBX1 expression level in piR-YBX1 OE and control TNBC cells. Scale bar: 25 μm. **G** Schematic illustration shows the wild-type binding site (pmirGLO-YBX1-wt) and mutated binding site (pmirGLO-YBX1-mut) in the dual-luciferase vectors. **H** Relative luciferase activity was measured after co-transfection of the piR-YBX1 mimic and the pmirGLO-YBX1-wt or pmirGLO-YBX1-mut vectors in TNBC cells, respectively. The data are showed as the mean ± SD, *P < 0.05 **P < 0.01, ***P < 0.001, ****P < 0.0001. Data were analyzed by (**C**, **D**) Students t test and (**H**) one-way ANOVA.

To probe the mechanism of piR-YBX1, we further extracted total RNA from three pairs of BT549 cells after transfection of piR-YBX1 mimic or piR-NC and conducted RNA-seq analysis to explore the potential gene targets of piR-YBX1(Additional file 5: Table S4). The top 20 changed genes were shown in the heatmap (Fig. 3A). Surprisingly, our data showed *YBX1* is the top downregulated gene in the list (Fig. 3B). There have been many studies exploring the function of YBX1 in cancers, and the majority of scholars believe that it is an oncogene [32, 33]. Additionally, our findings indicated that BC patients with elevated YBX1 expression have a poorer prognosis (Additional file 6: Fig. S2A). This observation was made through survival analysis using the Kaplan-Meier Plotter website (https://kmplot.com/analysis/). To verify whether *YBX1* is a target gene of piR-YBX1, we performed RT–qPCR analysis and observed that the expression of *YBX1* was markedly lower in the piR-YBX1 overexpression group than in the control group (Fig. 3C, D). Consistent with the result of mRNA expression, western blot revealed a corresponding reduction in YBX1 protein expression (Fig. 3E). To confirm the negative effect of piR-YBX1, an immunofluorescence assay was executed to find out the red fluorescence staining intensity of YBX1 expression. As expected, red fluorescence intensity was weaker in the piR-YBX1 overexpression group (Fig. 3F). To further study the interaction between YBX1 and piR-YBX1, we utilized bioinformatic analysis to predict the existence of binding sites between them in the piRNAQuestV.2 (http://dibresources.jcbose.ac.in/zhumur/pirnaquest2/start.php) databases. The results suggested that the entire length of piR-YBX1 can participate in complementary base pairing with nucleotides 569–595 in *YBX1* and the score was 225 points (with 170 points considered a passing score). To identify this conclusion, wild-type (pmirGLO-YBX1-wt) and mutant-type (pmirGLO-YBX1-mut) dual-luciferase receptor vectors containing the wild-type or mutant binding sequence of *YBX1* were constructed (Fig. 3G). We harvested cells after transfection of the piR-YBX1 mimic with the pmirGLO-NC, pmirGLO-YBX1-wt or pmirGLO-YBX1-mut plasmids to measure the dual-luciferase activity. It is obvious that piR-YBX1 mimic transfection strongly suppressed the luciferase activity in the pmirGLO-YBX1-wt group; however, no significant difference was observed in the mutant group (Fig. 3H). According to this study, piR-YBX1 binds directly with *YBX1* mRNA and enhances its degradation.

Taken together, these findings exposured that piR-YBX1 could bind to *YBX1* mRNA and inhibit YBX1 expression at both the transcript and protein levels.

### YBX1 mediates the proliferation and metastasis induced by piR-YBX1

**Fig. 4:**
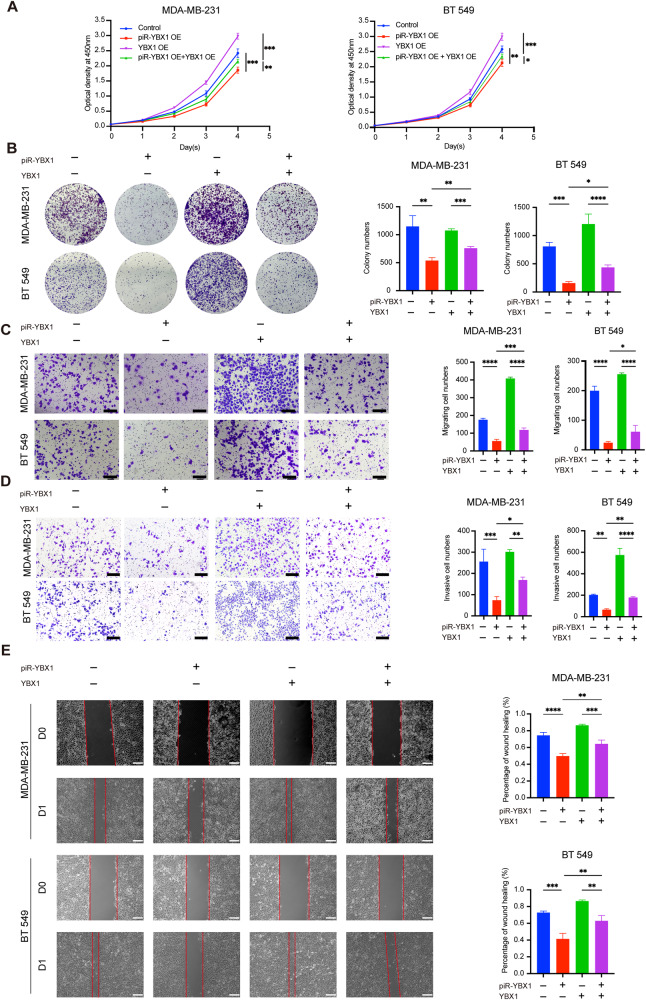
YBX1 mediates the proliferation and metastasis induced by piR-YBX1. **A** The proliferation of TNBC cells was measured after YBX1 OE in control and piR-YBX1 OE cells by CCK8. **B** YBX1 OE reduced the inhibited effect of piR-YBX1 on colony formation ability of TNBC cells. Representative pictures (left) and quantitative statistics (right) of colony numbers. **C, D** The effects of piR-YBX1 on migration and invasion were inhibited by YBX1 OE. Representative pictures (left) and quantitative statistics (right) of penetrating cells. Scale bar: 200 μm. **E** YBX1 OE reduced the inhibitory effect of piR-YBX1 on the wound healing ability of TNBC cells. Representative images (left) and quantitative statistics (right) of the migration rate. Scale bar: 100 μm. The data are showed as the mean ± SD, *P < 0.05 **P < 0.01, ***P < 0.001, ****P < 0.0001. Data were analyzed by (**A**) two-way ANOVA and (**B**–**E**) one-way ANOVA.

The above experiments demonstrated that piR-YBX1 can bind directly to *YBX1* mRNA and inhibit YBX1 expression. Next, we sought to investigate the role of YBX1 in TNBC cells growth and metastasis induced by piR-YBX1. Therefore, rescue experiments were conducted, and the transfected cells were grouped as follows: control group, piR-YBX1 OE group, YBX1 OE group and piR-YBX1 OE + YBX1 OE group. In the CCK8 and colony information assays, YBX1 overexpression partially abolished the negative proliferation effect of piR-YBX1 in MDA-MB-231 and BT549 cells (Fig. 4A, B). Besides, the inhibited effects of piR-YBX1 on migration and invasion could be reversed by YBX1 overexpression in the transwell migration and invasion assays (Fig. 4C, D). In the wound healing assay, migration rate of cells was dramatically reversed by YBX1 overexpression (Fig. 4E).

The findings presented in this section indicated that piR-YBX1 suppresses the proliferation, migration, and invasion abilities of TNBC cells via YBX1.

### piR-YBX1 restrains the MEK and ERK1/2 MAPK signaling pathway through YBX1

**Fig. 5:**
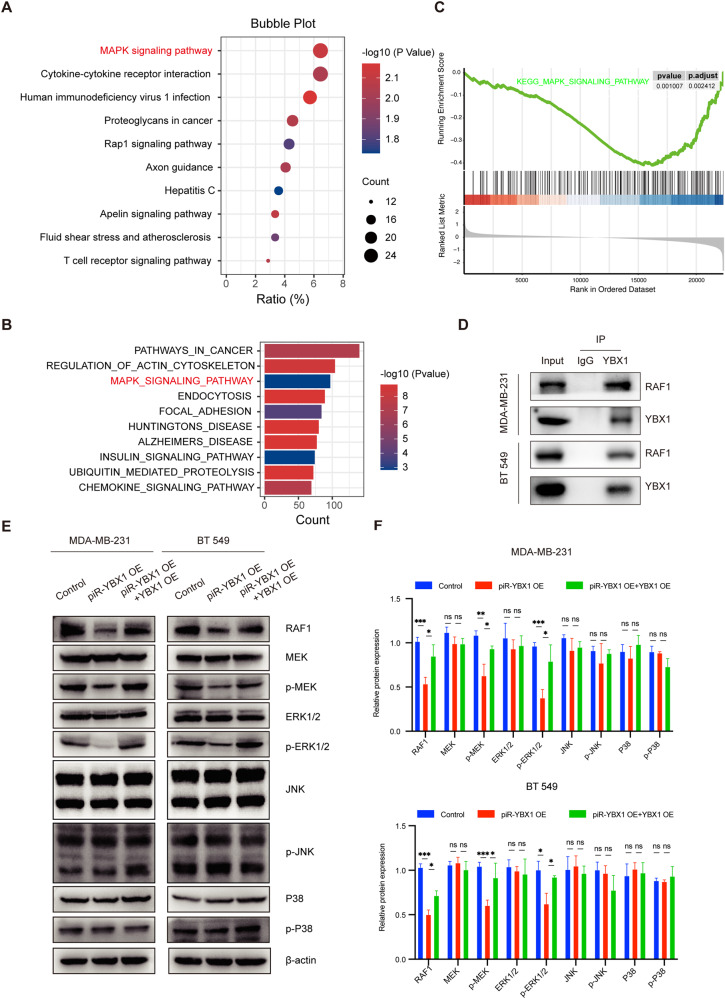
piR-YBX1 restrains the MEK and ERK1/2 MAPK signaling pathway through YBX1. **A** Bubble chart shows the result of KEGG enrichment analysis with the RNA-seq from piR-YBX1 OE and control cells. **B** Bar chart shows the results of GO analysis of piR-YBX1 group and control group. **C** ssGSEA analysis of genes related to the MAPK signaling pathway of *YBX1*. **D** Co-immunoprecipitation (Co-IP) experiments were carried out to investigate the interaction between YBX1 and RAF1 in TNBC cells. IP: mouse anti-YBX1 antibody; mouse-anti-IgG as a control. IB: rabbit anti-YBX1 and rabbit anti-RAF1. **E** Western blot shows that YBX1 reverted the effect of piR-YBX1 on the expression of p-MEK, p-ERK1/2 and other markers of the MAPK signaling pathway. **F** Western blot analysis of relative expression of protein level, protein levels are normalized to β-actin. The data are showed as the mean ± SD, *P < 0.05 **P < 0.01, ***P < 0.001, ****P < 0.0001. Data were analyzed by (**F**) one-way ANOVA.

To gain a deeper understanding of the molecular mechanisms by which piR-YBX1 makes its functions in TNBC cells, we use the RNA profiles from RNA-seq of BT549 cells, and KEGG pathway enrichment analysis and GO analysis was used to investigate the signaling pathways that piR-YBX1 associated with (Additional file 7: Table S5). As indicated by the bubble chart and bar chart, the MAPK signaling pathway was on the list of enriched pathways (Fig. 5A, B). Moreover, the MAPK signaling pathway was also enriched in single-sample gene set enrichment analysis (ssGSEA) for *YBX1* and most genes in the MAPK pathway were negatively correlated with it (Fig. 5C), suggesting that piR-YBX1 may influence the malignant phenotype of TNBC cells via the YBX1/MAPK axis.

MAPK is the abbreviation for mitogen-activated protein kinase, and MAPK signaling is hyperactivated in diverse types of tumors, which is typically considered to be associated with the initiation and metastasis of cancer [34, 35]. The MAPK pathway contains the MKKK-MKK-MAPK tertiary module, and the MAPKs include the ERK, P38 and JNK family members [36]. Targeting MAPK pathways has been regarded as a promising antitumor therapeutic strategy in recent years [37, 38]. Imada K [39] et al. discovered that YBX1 can interact with RAF1 and regulate its expression in prostate cancer. Therefore, we hypothesized that YBX1 can bind to RAF1 in TNBC cells and validated this hypothesis through an endogenous Co-IP assay, which demonstrated the connection between YBX1 and RAF1 (Fig. 5D). Then, we examined the MAPK pathway by western blot analysis using TNBC cells with YBX1 knockdown and discovered that YBX1 knockdown reduced the levels of p-MEK and p-ERK1/2 in the MAPK pathway (Additional file 8: Fig. S3A). Next, we analyzed the impact of piR-YBX1 on the MAPK pathway by western blot (Additional file 8: Fig. S3B). As anticipated, piR-YBX1 was responsible for the decreases of p-MEK and p-ERK1/2. It is worth noting that neither the total protein levels of MEK, ERK1/2, JNK, P38 nor the p-JNK and the p-P38 protein levels were significantly altered. Since YBX1 can rescue the negative effect induced by piR-YBX1, we further identified whether YBX1 accounts for the effect of piR-YBX1. The negative impact of piR-YBX1 was reverted by YBX1 overexpression in TNBC cells (Fig. 5E). Quantitative evaluation and statistical analysis of western blots are shown (Fig. 5F).

Overall, these findings proved that piR-YBX1 has the negative impact on the MAPK pathway which can be reversed by overexpression YBX1.

### piR-YBX1 inhibits tumor growth and lung metastasis of TNBC in vivo

**Fig. 6:**
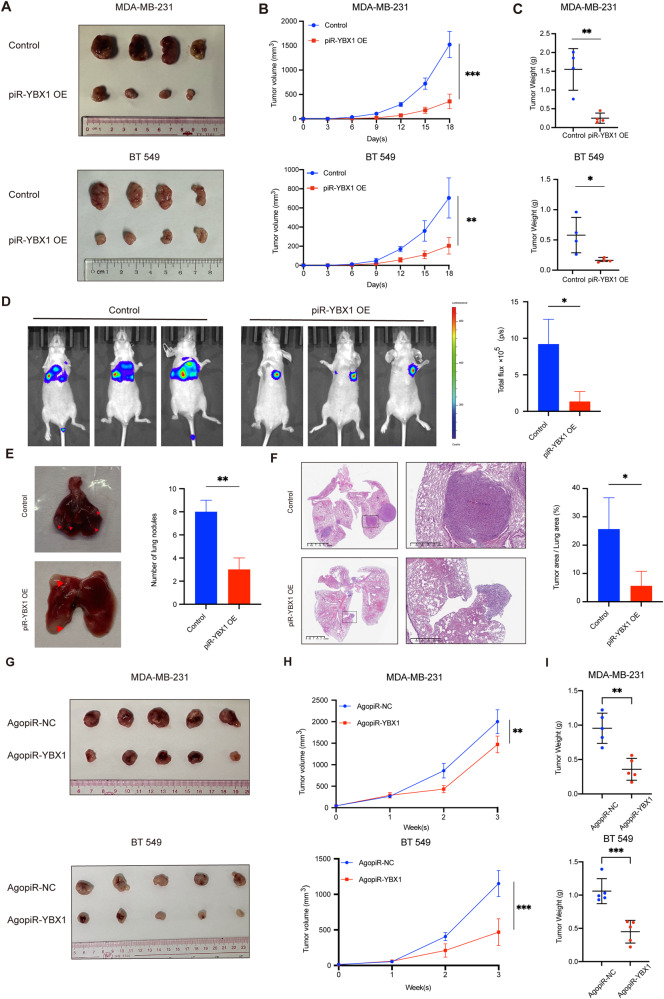
piR-YBX1 inhibits tumor growth and lung metastasis of TNBC in vivo. **A**–**C** Influence of piR-YBX1 on the growth of TNBC cells-derived tumors in mice. Tumor images, growth curves and tumor weights are shown. **D** Fluorescence images of mice show the antimetastatic effects of piR-YBX1 in vivo (left) and value for total flux of the lung metastasis ROI (right) is shown. **E** Representative pictures of macro-metastasis nodules in the lungs after intravenous injection of tumor cells (left) and statistics analysis of the number of macro-metastasis nodules in the lungs (right). **F** Presentative H&E staining photos of lung metastasis between control and piR-YBX1 OE group (left) and the percentage of tumor area in total lung area which shows the degree of lung metastasis. Scale bar: 2.5 mm. **G**–**I** One week after the injection of MDA-MB-231 and BT549 BC cells, agopiR-YBX1 and agopiR-NC were injected into the tumors. Tumor images, growth curves and tumor weights are shown. The data are showed as the mean ± SD, *P < 0.05 **P < 0.01, ***P < 0.001, ****P < 0.0001. Data were analyzed by (**B, H**) two-way ANOVA and (**C**–**F, I**) Students t test.

To further evaluate the function of piR-YBX1 in vivo, we injected MDA-MB-231 and BT549 cells stably overexpressing piR-YBX1 or a scrambled construct into the fat pads of female nude mice (11 ~ 17 g). The subcutaneous xenograft model showed that piR-YBX1 could also suppress the growth of tumors in vivo (Fig. 6A–C). Considering the impact on metastatic capability, we injected MDA-MB-23-luc-OE or MDA-MB-231-luc-NC cells via the tail vein and observe lung metastasis 7 weeks later. As shown by the pictures, the group injected with MDA-MB-231-luc-OE cells had smaller metastasis with a lower fluorescence value than the control group (Fig. 6D). By removing the lungs, we observed a reduction in the number of lung metastasis nodules in the group of mice injected with MDA-MB-231-luc-OE cells (Fig. 6E). Additional, H&E staining was performed on sections of lung to verify lung metastasis formation, and consistent with the overall results, the area of metastatic lesions in the piR-YBX1 overexpression group was significantly smaller than that in the control group, confirming that piR-YBX1 can inhibit lung metastasis of tumors (Fig. 6F). Furthermore, we performed immunohistochemistry on both mouse xenograft sections and lung metastatic lesions to observe the expression of YBX1. As shown in the figures, the expression of YBX1 was downregulated in both primary tumors and metastatic lesions (Additional file 9: Fig. S4A, B)

It is not clear if tumor-suppressive effects can still be achieved through direct administration of piR-YBX1 to mice, we used a chemically modified piR-YBX1 mimic called agopiR-YBX1 which can be existed in vivo conditions to detect its function [40]. Following the formation of xenografts, agopiR-YBX1 was injected into the tumors every two days for a total of 8 injections. Record tumor size weekly from the start of drug injection. After 3 weeks, the tumors were removed, and the volume of tumors treated with agopiR-YBX1 decreased compared with that of control tumors (Fig. 6G–I). All evidence showed that piR-YBX1 has the potential to be an effective agent for the treatment of cancer.

In a conclusion, piR-YBX1 has the potential to inhibit tumor growth and metastasis in vivo, which could have implications for clinical treatment.

## Discussion

**Fig. 7:**
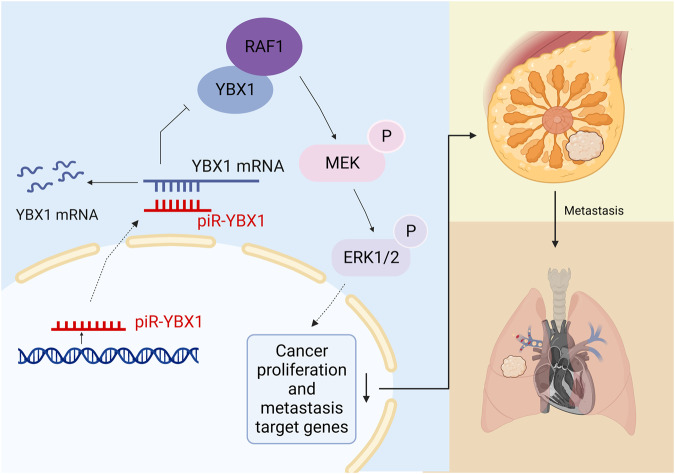
Schematic diagram of the mechanism of the piR-YBX1/YBX1/MAPK axis in TNBC. The figure is created with Biorender.com.

In this article, we identified piR-YBX1, a PIWI-interacting RNA, by piRNA microarray and confirmed that piR-YBX1 is downregulated in TNBC tissue in comparison to adjacent normal breast tissue. In addition, we were the first to explore the role of piR-YBX1 in BC. Overexpression of piR-YBX1 markedly decreased the growth and metastatic phenotype of TNBC cells. In addition, the same function of piR-YBX1 was recognized in vivo. We have reported that piR-YBX1 downregulates YBX1 expression by directly binding to *YBX1* mRNA and promoting its degradation. Besides that, mechanistic experiments suggested that piR-YBX1 regulates the MAPK signaling pathway via YBX1 (Fig. 7). We have also demonstrated that agopiR-YBX1 has the potential to be an effective agent due to its tumor-suppressive effect. This study examined how piR-YBX1 inhibits the growth and invasiveness of TNBC cells.

Although research on the biogenesis of piRNAs is increasing, it remains insufficient. Primary piRNAs are generated by piRNA clusters, which are transported into the cytoplasm and processed into mature piRNAs in the Yb body [41]. Typically, piRNAs can regulate target genes at three different levels: by transcriptional gene silencing, posttranscriptional gene silencing and binding to certain proteins and promoting multiprotein interactions [24]. An increasing amount of research has recently focused on the functions of piRNAs in cancer. piR-36712 may interact with the pseudogene SEPW1P RNA and reduce SEPW1P expression via miR-7 and miR-324, thereby inhibiting the development of BC [28]. Huiying Han [29] suggested that piRNA-30473 contributes to tumorigenesis and poor prognosis in diffuse large B-cell lymphoma by regulating m6A RNA methylation through WTAP, thereby triggering downstream signaling cascades. piR-14633 was observed to promote proliferation, migration, and invasion of cervical cancer cells by METTL14/CYP1B1 signaling axis [42]. piR-54265 interacts with PIWI2 to form the PIWI2/STAT3/p-SRC complex and promotes the malignant phenotype of colorectal cancer cells by increasing STAT3 phosphorylation. The concentration of piR-54265 in the serum of colorectal cancer patients is dependent on the stage of disease and is associated with sensitivity to chemotherapeutic agents; therefore, it can serve as a marker for prognosis and the response to chemotherapy [30]. In this study, the mechanism of a novel piRNA in TNBC was identified and clarified. The suppressive effect of piR-YBX1 on TNBC cells, along with the ability of agopiR-YBX1 to dramatically reduce the volume of xenografts in mice, suggests that piR-YBX1 may be used to treat TNBC patients in the future.

YBX1 is a member of the YBX family that binds to the YB box sequence in DNA [43]. It can bind to both nucleic acids and proteins to form complexes that regulate gene transcription, mRNA translation, and the packaging and stabilization of mRNA [44]. Numerous studies have revealed the oncogenic function of YBX1 [45, 46], and some ncRNAs can interact with YBX1 to influence the progression of cancer [47, 48]. The long noncoding RNA HUMT recruits YBX1 to form a complex and enhances the expression of FOX1 to promote lymph node metastasis of TNBC [49]. We determined that *YBX1* is a downstream target gene of piR-YBX1 using RNA-seq and discovered the direct link between them by dual-luciferase assays. To explore whether YBX1 is essential for the function of piR-YBX1, we conducted rescue experiments and found that YBX1 could partially reverse the phenotype caused by piR-YBX1. Other oncogenes in addition to YBX1 may be degraded by piR-YBX1, but more evidence is required to confirm this.

The MAPK pathway is one of the most important signaling pathways influencing the development of cancer [50], and it is related to not only the initiation of primary tumors but also to distant metastasis [51]. CircMAPK1 encodes a novel peptide named MAPK1-109aa that can suppress the phosphorylation of MAPK1 and inhibit the proliferation and metastasis of gastric cancer cells [52]. The RAF/MEK/ERK axis is active in over 40% of human cancers and is involved in physiological processes of cancer cells [53]. In this study, the MAPK signaling pathway was found by KEGG pathway enrichment analysis, and it was confirmed that piR-YBX1 overexpression inhibited the phosphorylation of MEK and ERK1/2. In prostate cancer, Kenjiro Imada [39] et al. found that YBX1 interacts with RAF1 and regulates its expression through proteasomal pathway. We exhibited that YBX1 can also bind to RAF1 and regulate the MAPK singling pathway in TNBC. Otherwise, YBX1 can revert the negative effects of piR-YBX1 via the MAPK pathway.

However, there are several limitations of this study. First, it is unknown whether other proteins are involved in the interaction between *YBX1* and piR-YBX1. The mechanism by which YBX1 binds to RAF1 and downregulates it was not examined. Furthermore, the effect of agopiR-YBX1 on the inhibition of distant metastasis was not proven.

## Conclusions

In this article, we identified a novel piRNA, piR-YBX1, which is downregulated in TNBC. piR-YBX1 substantially inhibited the proliferation and migration of TNBC cells by interacting with *YBX1* mRNA and inhibiting the MAPK pathway. In addition, agopiR-YBX1 effectively reduced the volume of xenografts in mice, suggesting that it has potential as a clinical agent for cancer treatment.

## Material and methods

### Study samples

All patient tissue samples were obtained from Sun Yat-Sen University Cancer Center (SYSUCC) Bio-bank. After removal from the patients, the tissues were immediately put into RNAlater (Invitrogen, USA) overnight and were stored at −80 °C until use. The study was approved by the ethics committees of Sun Yat-Sen University Cancer Center (IRB approval number: GZR2019-254).

### piRNA microarray analysis

Total RNA was extracted from TNBC tissue and paired adjacent normal breast tissue, RNA quantity and quality were measured using NanoDrop ND-1000. And piRNA microarray analysis was performed by Aksomics Inc (KangChen Bio-tech, Shanghai, China). The Arraystar HG19 piRNA array is designed for profiling of piRNAs in human germ cells. Human piRNAs are downloaded from NCBI database and mapped to the HG19 genome sequence using UCSC Blat. Our original data has been uploaded to the Gene Expression Omnibus (GEO) database with the accession number GSE 245714.

### Cell lines and culture

All BC cells were purchased from the American Type Culture Collection and all cell lines were authenticated by profiling. All cell lines were cultured in DMEM (Gibco, USA) supplemented with 10% FBS (Excell, China) and 1% penicillin-streptomycin (Gibco, USA). All cells grown in a humidified incubator at 37 °C with 5% CO2.

### RNA extraction and quantitative real-time polymerase chain reaction (RT–qPCR)

Total RNA was extracted from cells using an RNA-Quick Purification Kit (ESscience, China). Reverse transcription was performed with random or specific piRNA stem-loop reverse transcription primers using PrimerScript RT Master Mix (Takara, USA) according to the manufacturer’s protocol, and 500 ng RNA was used. RT–qPCR was used to determine the RNA levels using TB Green Premix Ex Taq (Takara, USA) in a Bio-Rad CFX96. Relative RNA expression was normalized to that of GAPDH or U6 RNA using the comparative Ct method. The primer sequences are shown in Additional file 1: Table S1.

### Plasmids, transfection, and lentiviral transduction

The pcDNA3.1(+)-YBX1-HA plasmid containing the HA tag for YBX1 overexpression, and the pmirGLO-YBX1-wt, pmirGLO-YBX1-mut, and pmirGLO-NC plasmids were purchased from Umine Biotechnology Co.,Ltd (Guangzhou, China). In addition, YBX1 siRNAs were purchased from GenePharma (Suzhou, China). The piR-YBX1 mimic and the negative control mimic, agopiR-YBX1 and agopiR-NC were designed and synthesized by GenePharma (Suzhou, China). Both plasmids and siRNAs were transfected wtih Lipofectamine 3000 (Invitrogen, USA) according to the manufacturer’s instructions. The siRNAs, mimics and agopiRs sequences are listed in Additional file 2: Table S2.

The lentiviral plasmid overexpressing piR-YBX1 was purchased from Vigene Biosciences (Shandong, China), and empty vector was used as a control. TNBC cells were infected with lentivirus and selected with puromycin (1 µg/ml) after 24 h. The expression of piR-YBX1 was proven by RT–qPCR.

### Cell proliferation assays

For cell proliferation assays, cells were seeded in 96-well plates, and each well contained 1000 cells and 100 µl of culture medium. After cell adherence, the culture medium was replaced according to the instructions of the CCK8 kit (GLPBIO, USA) and incubated for 2 h, and the spectrophotometric absorbance at 450 nm was then measured every 24 h. For colony formation assays, 1000 cells were seeded in 6-well plates. After incubation for 14 days, the cells were fixed with methanol and stained with 0.1% crystal violet.

### Migration and invasion assays and wound healing assays

Migration assays were conducted in 8-μm pore inserts (Corning, USA) and 24-well plates. MDA-MB-231 (2 × 10^4^) and BT549 (4 × 10^4^) cells were suspended in 200 μl of FBS-free DMEM and seeded in the upper chambers, and 700 μl of DMEM with 20% FBS was added to the lower chambers. Cells were fixed with methanol and stained with 0.1% crystal violet after 20 h of incubation, and the cells remaining in the upper chambers were removed. For invasion assays, cells (4 × 10^4^ MDA-MB-231 cells per well and 6 × 10^4^ BT549 cells per well) were seeded in the upper chambers in which the membrane was coated with Matrigel (Corning, USA), and the other procedures were the same as those used for the migration assays.

When cells in the 6-well plate were 90% confluent, a micropipette tip was used to scratch the cell layer in a straight line to form a wound. The plates were imaged at 0 h and 18 h (MDA-MB-231) or 48 h (BT549) under a microscope.

### EdU (5-ethynyl-2′-deoxyuridine) incorporation assay

Cells were incubated in 24-well plates, and an EdU Kit (Beyotime, China) was used following the manufacturer’s protocols. DAPI was used to stain nuclei. Images of EdU-labeled cells and total cells were acquired by fluorescence microscopy, and the cells were counted by ImageJ.

### Western blot analysis

Cells were lysed and proteins were extracted on ice for 10 min with RIPA lysis buffer (EcoTop, China). Then, the proteins were boiled at 100 °C for 10 min with 5× loading buffer (FDbio, China). Proteins were separated by SDS-PAGE and were then transferred to 0.45 μm PVDF membranes. The membranes were blocked in 5% nonfat milk (for analysis of unphosphorylated proteins) or 5% BSA (for analysis phosphorylated proteins) before they were incubated with primary antibody. The membranes were incubated with secondary antibody the next day. The blots were visualized by an FDBio-Dura ECL Kit (FDbio, China). The antibodies are listed in Additional file 3: Table S3. The data of western blot was quantitatively analyzed by ImageJ.

### Co-immunoprecipitation assays

Proteins were harvested from TNBC cells to perform endogenous Co-IP. We extracted proteins on ice for 30 min with IP lysis buffer (Beyotime, China). The lysate was concentrated by centrifugation at 14,000 × g for 10 min, and the supernatant was then divided into three aliquots. One aliquot was directly added 5× loading buffer, boiled for 10 min at 100 °C, and then stored at −20 °C as the “Input”. The other two aliquots were incubated with an antibody (anti-YBX1, mouse or anti-IgG, mouse) at 4 °C overnight and were then incubated with protein A/G beads (MCE, USA) with rotation for 1 h at room temperature the next day. The immunoprecipitated samples were washed 6 times with IP lysis buffer for 5 min each. The immunoprecipitated samples were added to 1× loading buffer (Beyotime, China) and boiled for 10 min at 100 °C before proteins separation by SDS-PAGE.

### Dual-luciferase reporter gene assays

Cells were harvested 48 h after cotransfection of the YBX1 mimic and pmirGLO plasmids. The cells were lysed, and the luciferase activity of lysate was measured using a Dual Luciferase Reporter Gene Assay Kit (Yeasen, China).

### Immunofluorescence

Autoclaved glass slides were placed in 24-well plates, and the appropriate cell suspension was then added until the cell density was about 50–70%. After three washes, the cells were fixed with ice-cold ethanol for 10 min and were washed three times again. Then, 0.1% Triton X-100 in PBS was used to permeabilize the cells, and the slides were washed. The cells were incubated with primary antibody overnight at 4 °C after blocking with 5% BSA for 2 h. Afterwards, the cells were incubated with fluorescent secondary antibody for 1 h in the dark. The secondary antibody was removed, and DAPI was added to stain nuclei. Images were acquired with a fluorescence microscope.

### Immunohistochemistry

Immunohistochemistry was performed according to standard protocols. Paraffin sections were dewaxed, and rehydrated, endogenous peroxidase activity was blocked, and the sections were incubated with primary antibody overnight at 4 °C. The sections were washed and incubated with a horseradish peroxidase-labeled polymer for 25 min. Then, the sections were stained with DAB and then counterstained with hematoxylin. Images were taken using an inverted microscope.

### Hematoxylin and eosin (H&E) staining

The lung tissue was fixed in 4% formaldehyde, embedded in paraffin, and subsequently sectioned into slices. Following dewaxing and rehydration, the sections were immersed in hematoxylin for 5 min before being rinsed under running water for 2 min. After differentiation and eosin staining, the sections were dehydrated, sealed, and observed under a microscope.

### Animal experiments

All animal experiments were approved by the Animal Ethics Committee of Sun Yat-sen University Cancer Center (IACUC ID: L102012019007W). We purchased BALB/c nude mice aged 3–4 weeks from Beijing Vital River Laboratory Animal Technology Co., Ltd. The nude mice were randomly divided into two groups. Experiments were begun after the mice had adapted to the new environment for 1 week. For the growth assays, 1 × 10^7^ of MDA-MB-231 and BT549 cells with stable piR-YBX1 overexpression or corresponding control cells were washed with PBS one time and then diluted with 200 μl PBS, after which cells were injected into the fat pad of mice (n = 4/group). Beginning on Day 6, tumor size was measured every 3 days for tumor growth curve, and after 18 days, we surgically resected the tumors from the mice for analysis. For the metastasis assay, 2 × 10^6^ luciferase labeled MDA-MB-231-luc-OE (stable overexpressing piR-YBX1) cells or MDA-MB-231-luc-NC (control group) cells diluted with 60 μl PBS were injected into 5-week-old mice via the tail vein (n = 3/group). Seven weeks later, the mice were injected intraperitoneally with luciferin (Promega) to observe lung metastasis using a Living Image® system (Perkin Elmer) and the quantitative data were expressed as photon flux. To examine the clinical role of piR-YBX1, 1 nmol AgopiR-YBX1 (GenePharma, Suzhou, China) or AgopiR-NC (GenePharma, Suzhou, China) dissolved in 50 μl PBS was injected into the mouse tumors twice a day after injecting 1 × 10^7^ of MDA-MB-231 and BT549 cells 7 days to study the role of piR-YBX1 in animals.

### Statistical analysis

Statistical analysis was performed with Prism GraphPad 9.0.2 software. Differences between two groups of independent samples were analyzed by Students t test, and differences among more than two groups were analyzed by one-way ANOVA. Two-way ANOVA was used for the analysis of data with more than one independent variable. The experiments were performed at least three times independently. *P* < 0.05 indicates statistical significance.

### Supplementary information

Additional file 1 Table S1

Additional file 2 Table S2

Additional file 3 Table S3

Additional file 4 Supplementary Fig. S1

Additional file 5 Table S4

Additional file 6 Supplementary Fig. S2

Additional file 7 Table S5

Additional file 8 Supplementary Fig. S3

Additional file 9 Supplementary Fig. S4

Additional file 10 Original data of western blot

## Data Availability

All data generated during this study are included in this published article.
