# Supplementary material for: PIWI-interacting RNA-YBX1 inhibits proliferation and metastasis by the MAPK signaling pathway via YBX1 in triple-negative breast cancer
Source: Cell Death Discov. 2024 Jan 5;10:7. doi: 10.1038/s41420-023-01771-w (PMC10770055; doi:10.1038/s41420-023-01771-w)
Supplement: Supplementary file 1 — Additional file 1 Table S1 [file 41420_2023_1771_MOESM1_ESM.docx]

**Supplementary Table S1.** Primers used for RT-qPCR in this study.

| Gene Symbol | Forward primer (5’→3’) | Reverse primer (5’→3’) |
| --- | --- | --- |
| YBX1 | TAGACGCTATCCACGTCGTAG | ATCCCTCGTTCTTTTCCCCAC |
| GAPDH | GAAGGTGAAGGTCGGAGTC | GAAGATGGTGATGGGATTTC |
| U6 | CTCGCTTCGGCAGCACA | AACGCTTCACGAATTTGCGT |
| β-actin | CAGGGCGTGATGGTGGGCATG | GTAGAAGGTGTGGTGCCAGATT |

| Primers used for stem-loop RT-PCR of piRNAs | |
| --- | --- |
| Product Number | Product name |
| ssD089261711 | Bulge-LoopTM miR-Reverse Primer |
| miR8006931 | Bulge-LoopTM h-piRNA-YBX1 Forward Primer |
| miR8006930 | Bulge-LoopTM h-piRNA-YBX1 RT Primer |
| ssD0904071006 | Bulge-LoopTM U6-Forward Primer |
| ssD0904071007 | Bulge-LoopTM U6-Reverse Primer |
| ssD0904071008 | Bulge-LoopTM U6-RT Primer |
