# Supplementary material for: PIWI-interacting RNA-YBX1 inhibits proliferation and metastasis by the MAPK signaling pathway via YBX1 in triple-negative breast cancer
Source: Cell Death Discov. 2024 Jan 5;10:7. doi: 10.1038/s41420-023-01771-w (PMC10770055; doi:10.1038/s41420-023-01771-w)
Supplement: Supplementary file 2 — Additional file 2 Table S2 [file 41420_2023_1771_MOESM2_ESM.docx]

**Supplementary Table S2.** Sequences of synthesized siRNAs, mimics and agopiRs used in this study.

| Sequences of small interfering RNAs | |
| --- | --- |
| YBX1-siRNA#1 | 5’- CAGTTCAAGGCAGTAAATATGCA -3’ |
| YBX1-siRNA#2 | 5’- GACGGCAATGAAGAAGATAA -3’ |
| Sequences of piR-YBX1 mimics | |
| piR-YBX1 mimic-sense | 5’-UGGAUAGCGUCUAUAAUGGUUACGGUC-3’ |
| piR-YBX1 mimic-antisense | 5’-CCGUAACCAUUAUAGACGCUAUCCAUU-3’ |
| Negative control mimic-sense | 5’-UUCUCCGAACGUGUCACGUTT-3’ |
| Negative control mimic-antisense | 5’-ACGUGACACGUUCGGAGAATT-3’ |
| Sequences of AgopiR-YBX1 used for in vivo assay | |
| AgopiR-NC-sense | 5'chol- UUCUCCGAACGUGUCACGUTT(2'OMe)-3' |
| AgopiR-NC-antisense | 5'chol- ACGUGACACGUUCGGAGAATT(2'OMe)-3' |
| AgopiR-YBX1-sense | 5'chol- UGGAUAGCGUCUAUAAUGGUUACGGUC(2'OMe)-3' |
| AgopiR-YBX1-antisense | 5'chol-CCGUAACCAUUAUAGACGCUAUCCAUU(2'OMe)-3' |
