# Supplementary material for: PIWI-interacting RNA-YBX1 inhibits proliferation and metastasis by the MAPK signaling pathway via YBX1 in triple-negative breast cancer
Source: Cell Death Discov. 2024 Jan 5;10:7. doi: 10.1038/s41420-023-01771-w (PMC10770055; doi:10.1038/s41420-023-01771-w)
Supplement: Supplementary file 3 — Additional file 3 Table S3 [file 41420_2023_1771_MOESM3_ESM.docx]

**Supplementary Table S3.** Antibodies used in this study.

Primary antibodies

| Gene Symbol | Brand | Host / Isotype | Cat Number |
| --- | --- | --- | --- |
| YBX1  YBX1 | Proteintech  Santa Cruz | Rabbit / IgG  Mouse / IgG | 20339-1-AP  sc-398340 |
| RAF1 | ABclonal | Rabbit / IgG | A19638 |
| MEK | ABclonal | Rabbit / IgG | A4868 |
| p-MEK | ABclonal | Rabbit / IgG | AP1349 |
| ERK1/2 | ABclonal | Rabbit / IgG | A4782 |
| p-ERK1/2 | ABclonal | Rabbit / IgG | AP0974 |
| JNK | ABclonal | Rabbit / IgG | A4867 |
| p-JNK | ABclonal | Rabbit / IgG | AP1337 |
| P38 | ABclonal | Rabbit / IgG | A4771 |
| p-P38 | ABclonal | Rabbit / IgG | AP0526 |
| β-actin | ABclonal | Rabbit / IgG | AC026 |
| GAPDH | Affinity | Rabbit / IgG | AF7021 |
| HA-tag | Proteintech | Mouse / IgG1 | 66006-2-Ig |
| IgG | Mouse IgG | Female Balb/C mice | B900620 |

Secondary antibodies

| Product Name | Brand | Cat Number |
| --- | --- | --- |
| Goat Anti-Rabbit IgG Antibody (H+L), HRP Conjugated | Bioss | bs-0295G-HRP |
| Alkaline Phosphatase AffiniPure Goat Anti-Mouse IgG (H+L) | Yeasen | 33202ES60 |
| CoraLite594 – conjugated Goat Anti-Rabbit IgG(H+L) | Proteintech | SA00013-4 |
