# Supplementary material for: PIWI-interacting RNA-YBX1 inhibits proliferation and metastasis by the MAPK signaling pathway via YBX1 in triple-negative breast cancer
Source: Cell Death Discov. 2024 Jan 5;10:7. doi: 10.1038/s41420-023-01771-w (PMC10770055; doi:10.1038/s41420-023-01771-w)
Supplement: Supplementary file 4 — Additional file 4 Supplementary Fig. S1 [file 41420_2023_1771_MOESM4_ESM.docx]

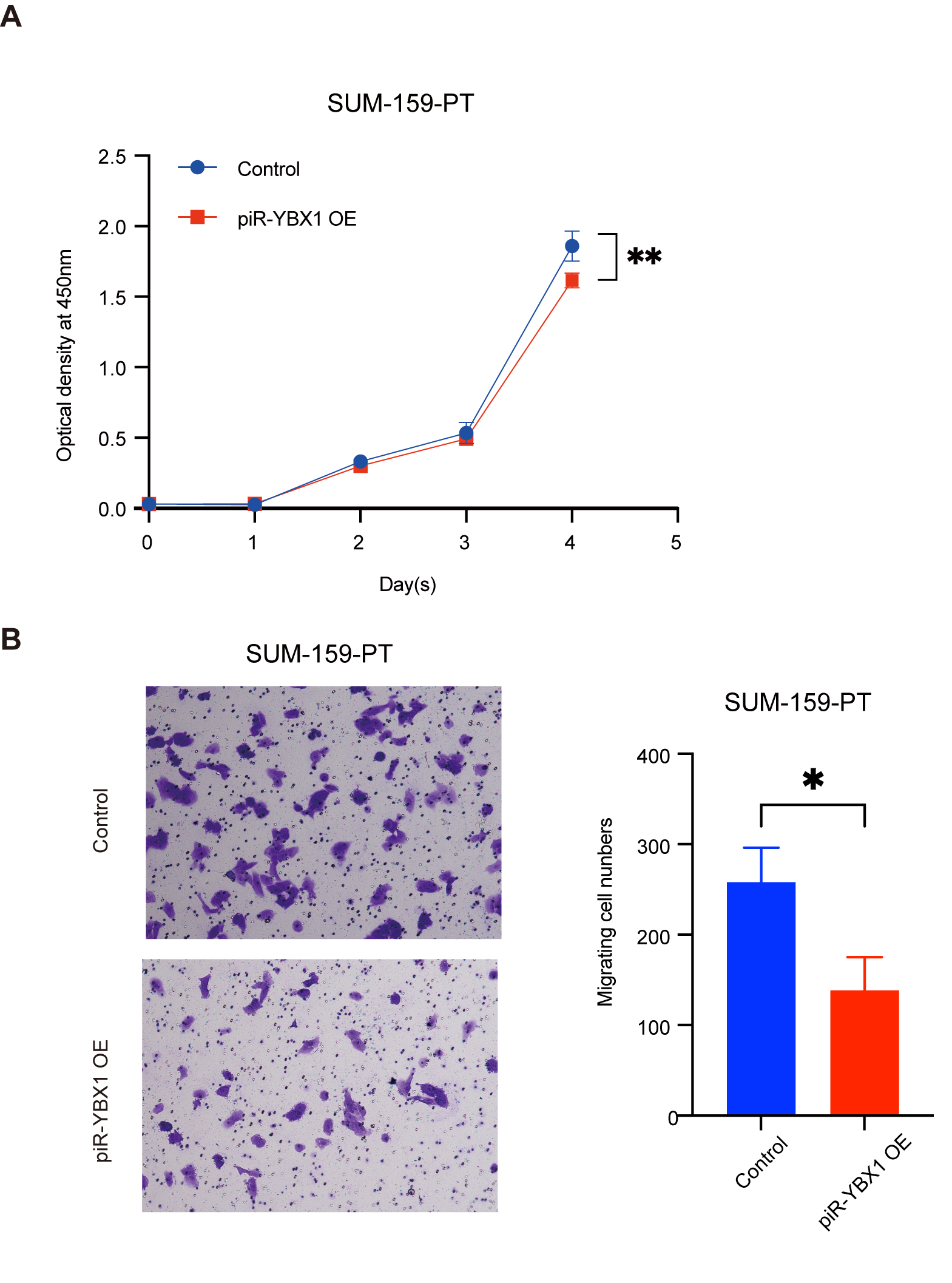


Fig. S1. **A** Overexpression of piR-YBX1 in SUM-159-PT cells inhibited their proliferation. **B** The negative effect of piR-YBX1 on the migration of SUM-159-PT cells. The data are showed as the mean ± SD, *P < 0.05 **P < 0.01, ***P < 0.001, ****P < 0.0001. Data were analyzed by (A) two-way ANOVA and (B) Students t test.
