# Supplementary figures and images for: PIWI-interacting RNA-YBX1 inhibits proliferation and metastasis by the MAPK signaling pathway via YBX1 in triple-negative breast cancer
Source: Cell Death Discov. 2024 Jan 5;10:7. doi: 10.1038/s41420-023-01771-w (PMC10770055; doi:10.1038/s41420-023-01771-w)

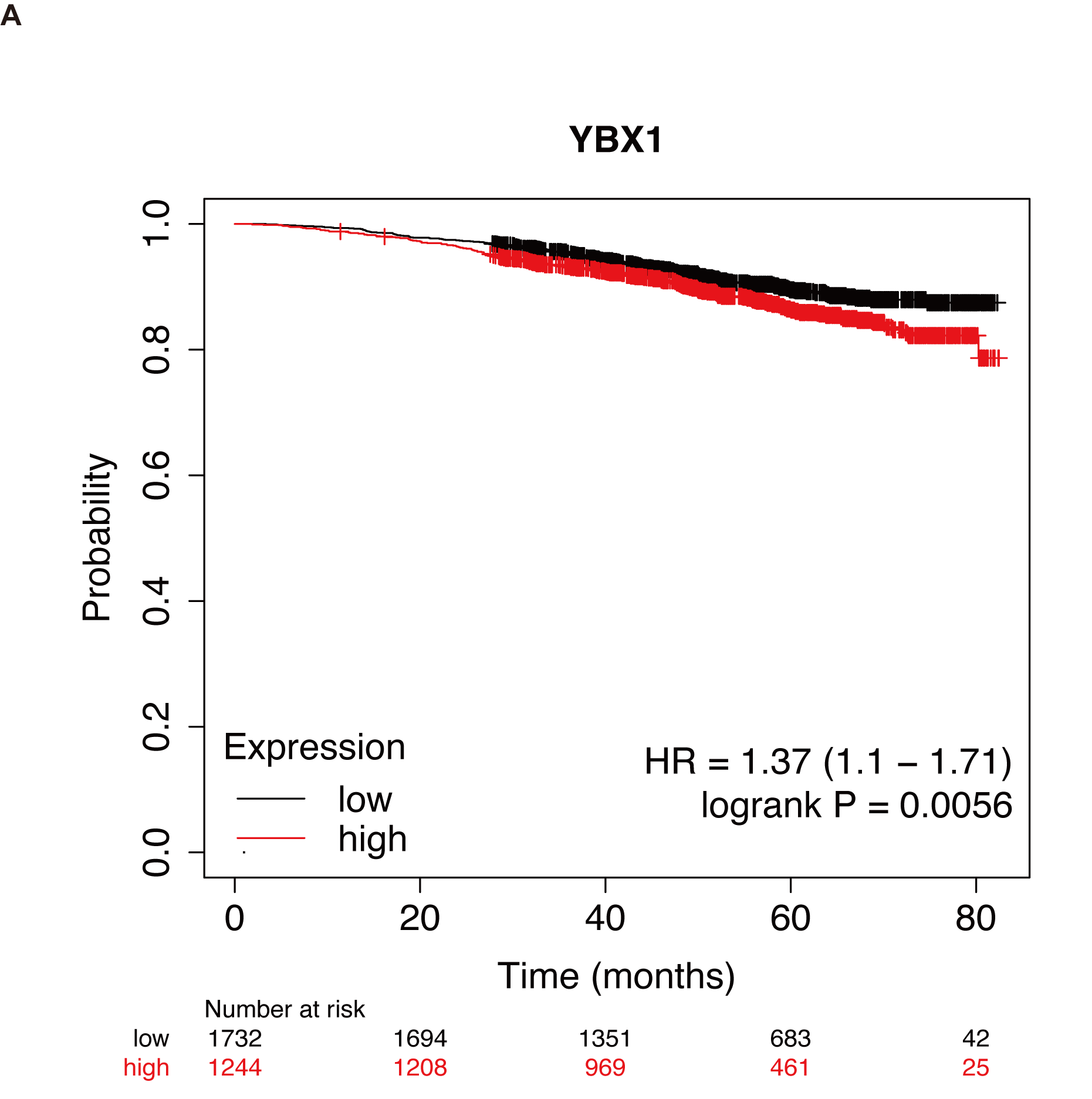


Fig. S2 **A** The prognosis of BC patients with high expression of YBX1 is poor.

Supplement: Supplementary file 6 — Additional file 6 Supplementary Fig. S2 [file 41420_2023_1771_MOESM6_ESM.docx]

Fig.2

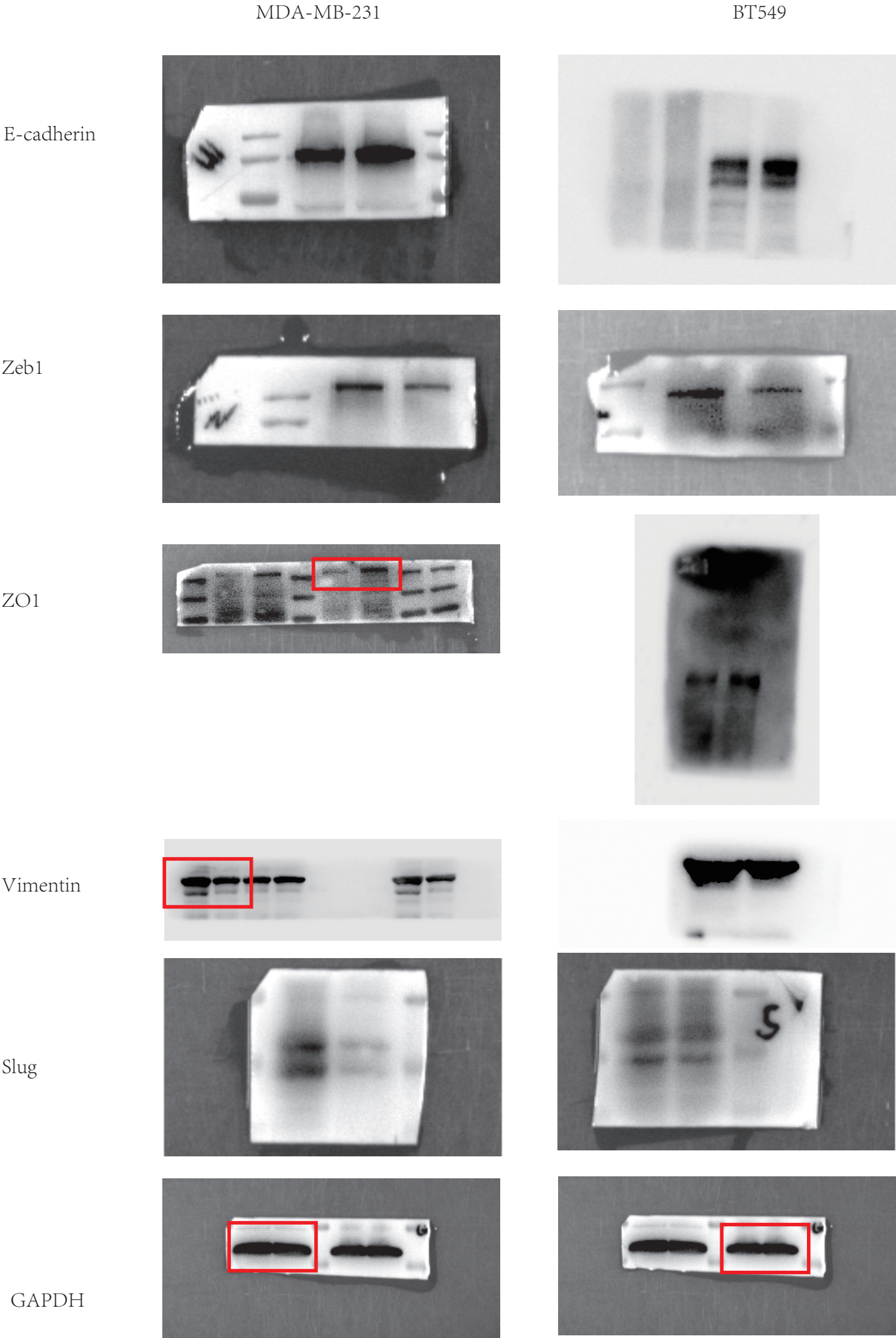

Fig.3

MDA-MB-231                      BT549

YBX1

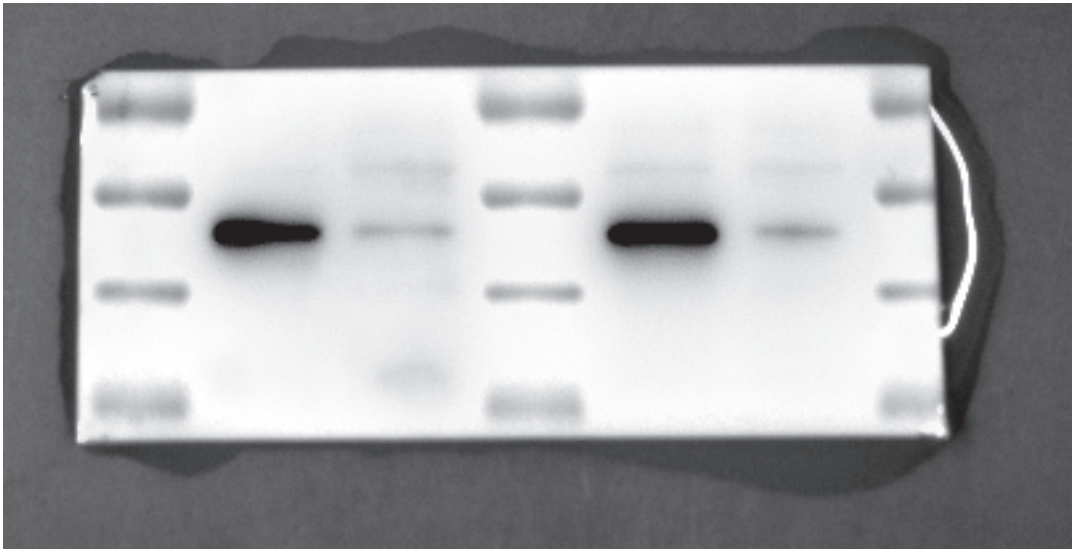

GAPDH

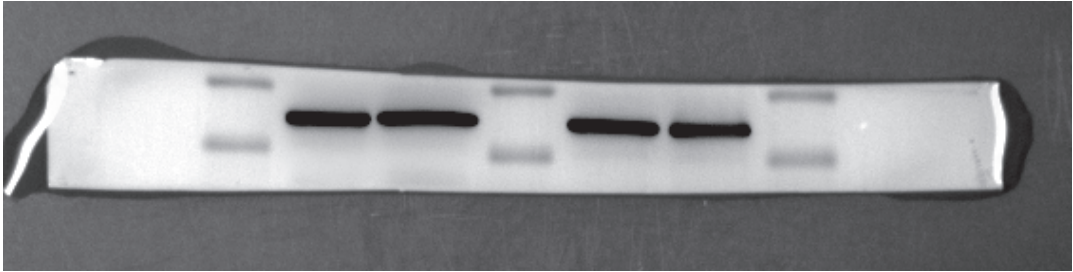

Fig.5D

MDA-MB-231

BT549

RAF1

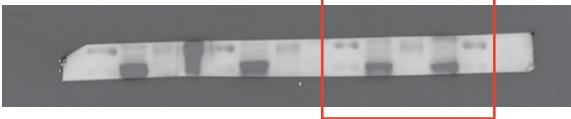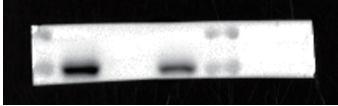

YBX1

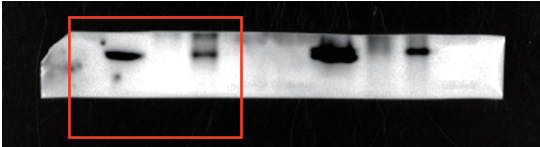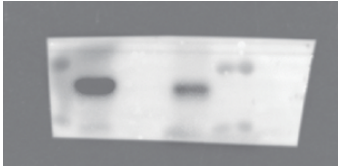

Fig.5E

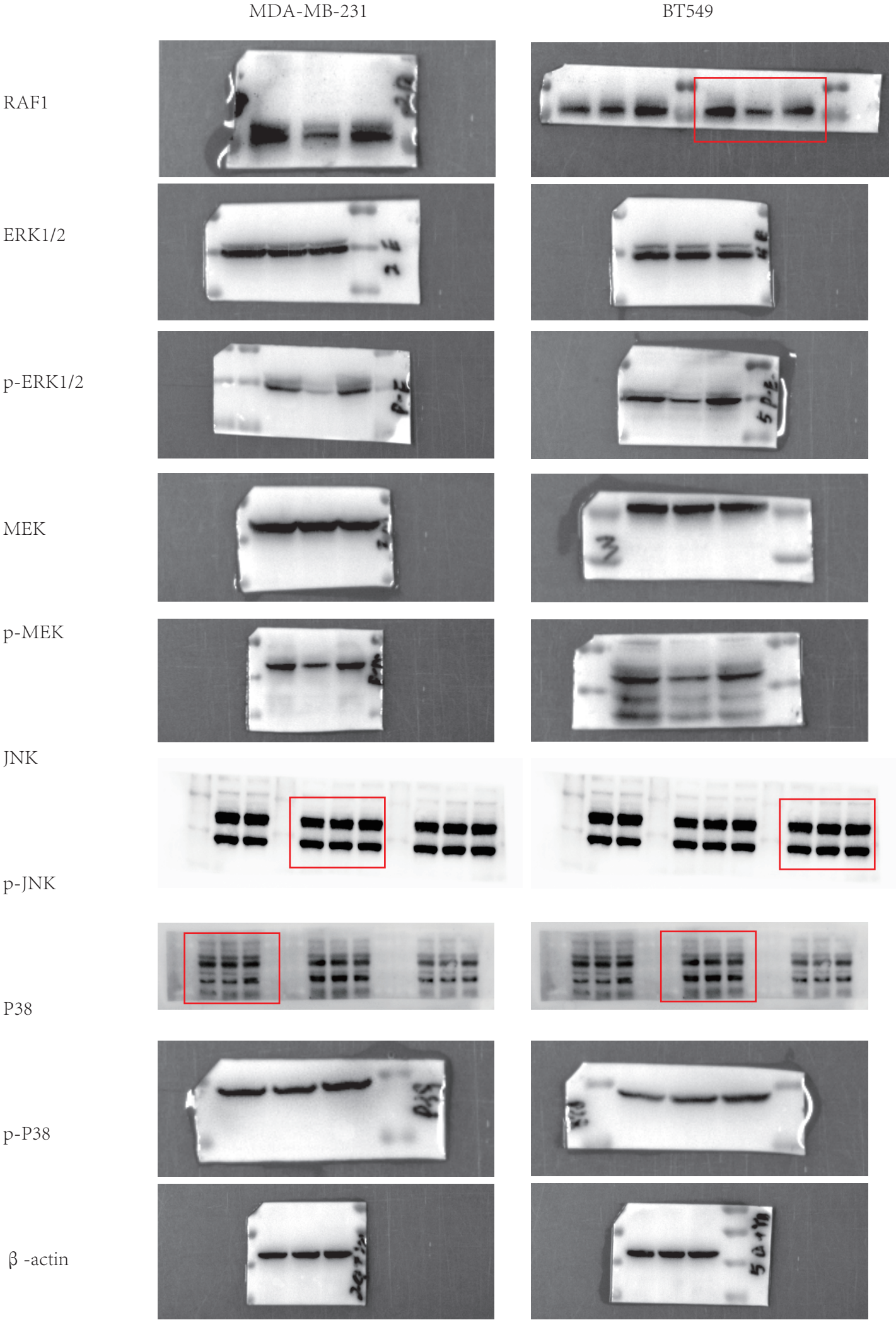

Fig.S3A

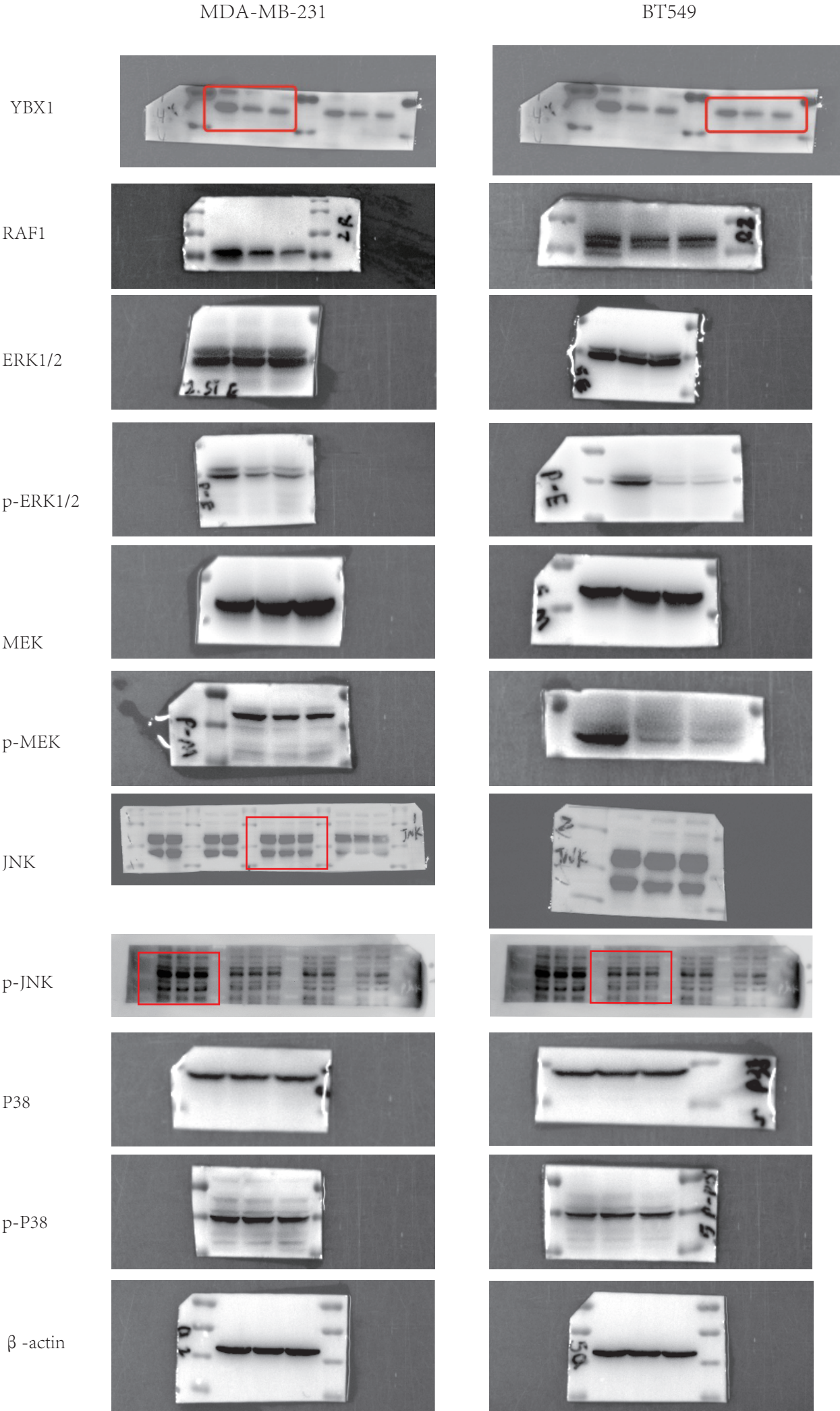

Fig.S3B

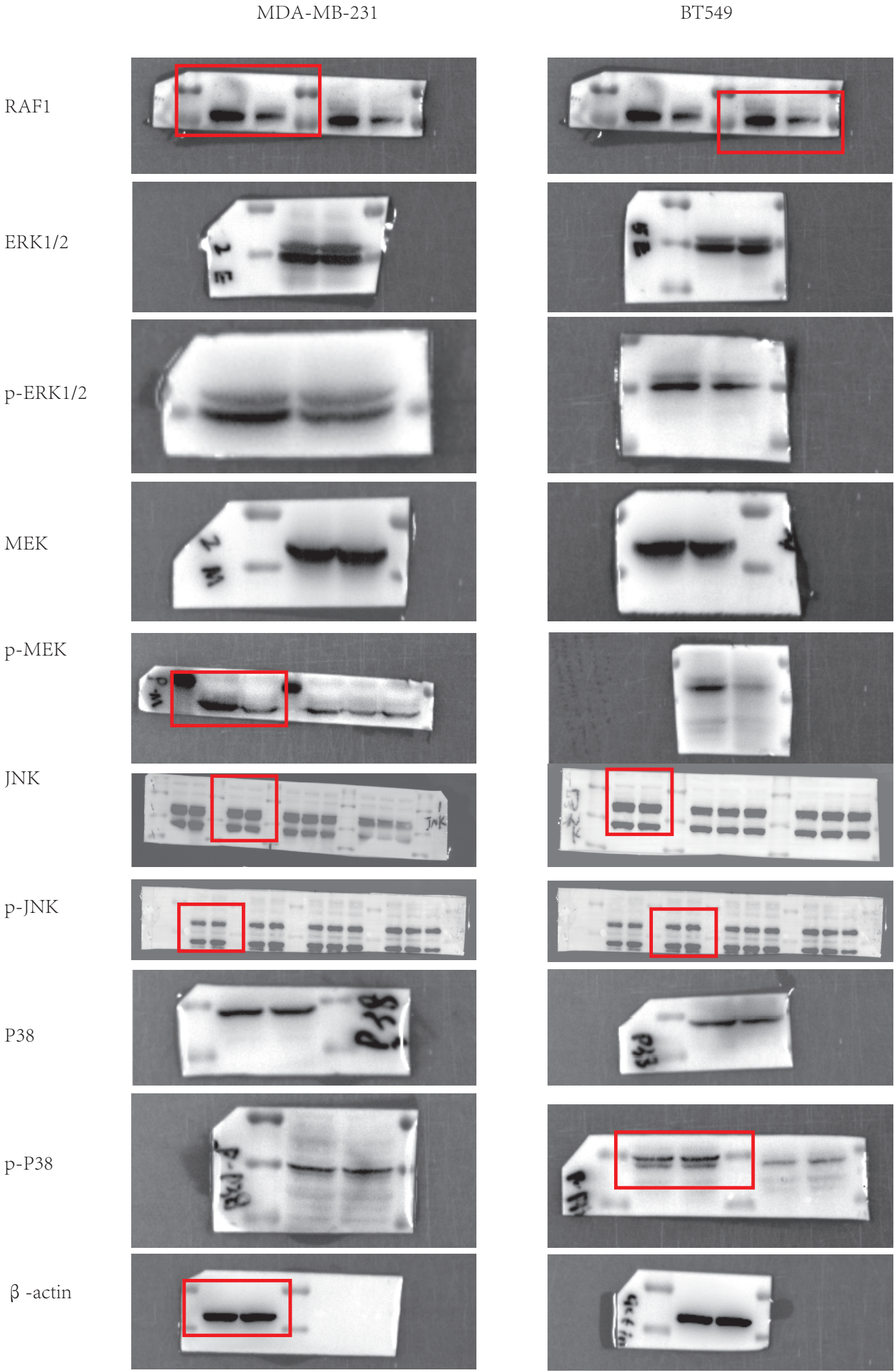

Supplement: Supplementary file 10 — Additional file 10 Original data of western blot [file 41420_2023_1771_MOESM10_ESM.pdf]
