# Supplementary material for: PIWI-interacting RNA-YBX1 inhibits proliferation and metastasis by the MAPK signaling pathway via YBX1 in triple-negative breast cancer
Source: Cell Death Discov. 2024 Jan 5;10:7. doi: 10.1038/s41420-023-01771-w (PMC10770055; doi:10.1038/s41420-023-01771-w)
Supplement: Supplementary file 8 — Additional file 8 Supplementary Fig. S3 [file 41420_2023_1771_MOESM8_ESM.docx]

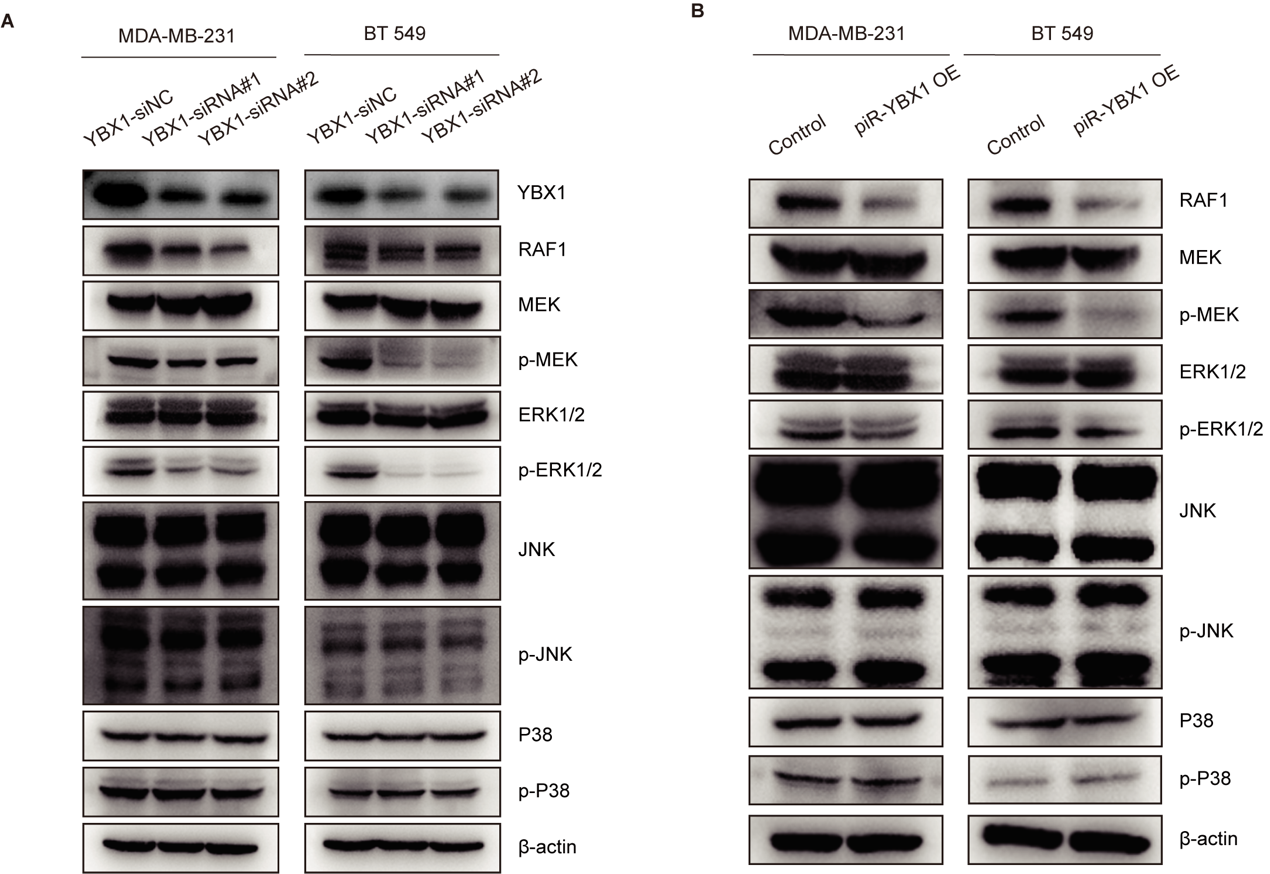


Fig. S3. **A** Knockdown of YBX1 in MDA-MB-231 and BT549 cells decreased the protein levels of RAF1, p-MEK and p-ERK1/2 in TNBC cells. **B** Western blot shows the changes of RAF1, p-MEK, p-ERK1/2 and other markers of the MAPK signaling pathway in piR-YBX1 OE TNBC cells.
