# Supplementary material for: PIWI-interacting RNA-YBX1 inhibits proliferation and metastasis by the MAPK signaling pathway via YBX1 in triple-negative breast cancer
Source: Cell Death Discov. 2024 Jan 5;10:7. doi: 10.1038/s41420-023-01771-w (PMC10770055; doi:10.1038/s41420-023-01771-w)
Supplement: Supplementary file 9 — Additional file 9 Supplementary Fig. S4 [file 41420_2023_1771_MOESM9_ESM.docx]

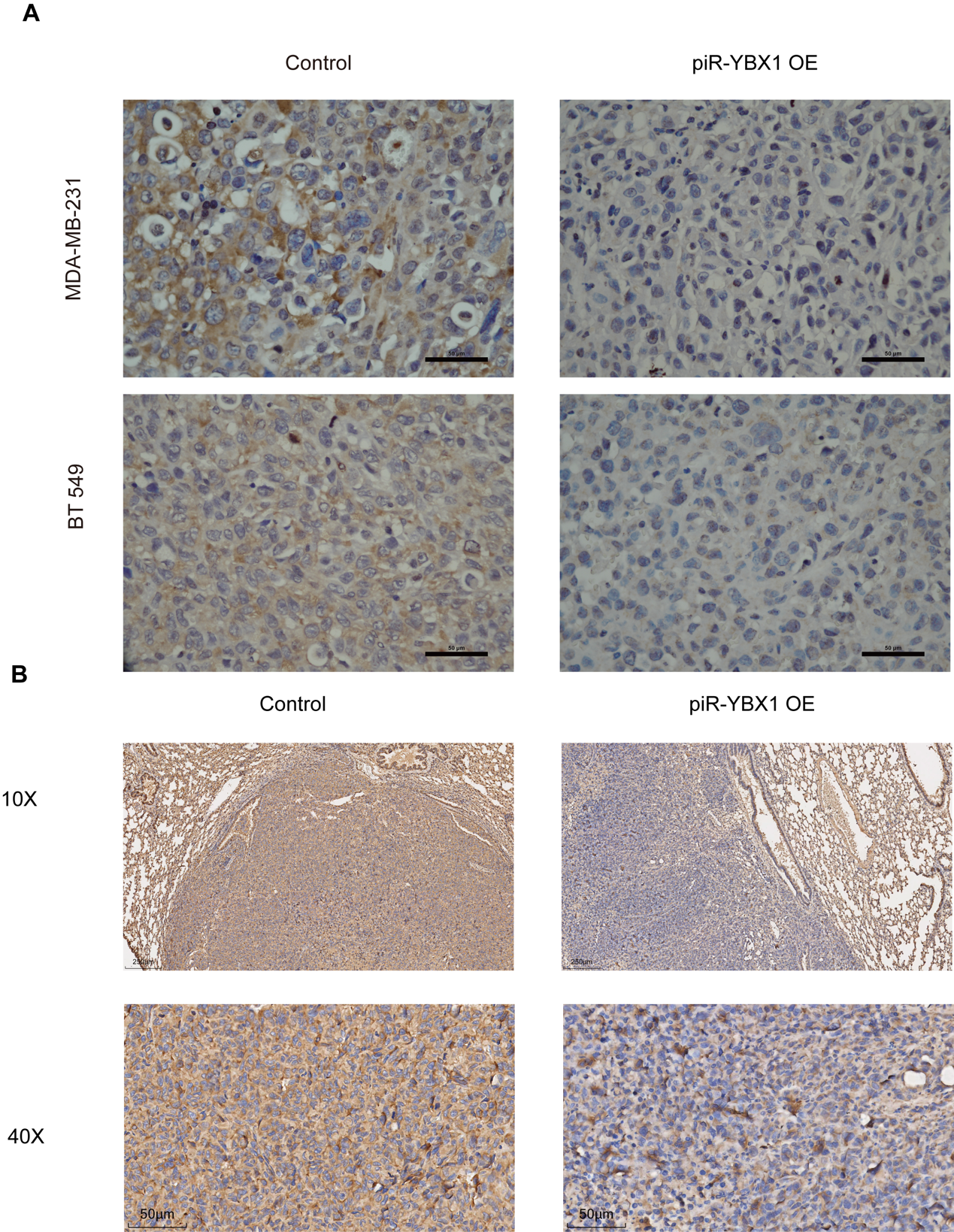


Fig. S4. **A** Immunohistochemical staining was used to analyze the expression of YBX1 in mouse xenograft tumors. Scale bar: 50μm.**B** Immunohistochemical staining of mouse lung metastasis sections revealing the expression level of YBX1. Scale bar: 250μm(10×), 50μm(40×).
